# Relapse risk prediction in patients with first-episode bipolar disorder: development, external validation, and pharmacotherapy associations of a machine learning model

**DOI:** 10.1038/s41380-025-03316-2

**Published:** 2025-10-23

**Authors:** Johannes Lieslehto, Jari Tiihonen, Markku Lähteenvuo, Alexander Kautzky, Aemal Akhtar, Ridwanul Amin, Bergný Ármannsdóttir, Stefan Leucht, Christoph U. Correll, Ellenor Mittendorfer-Rutz, Antti Tanskanen, Heidi Taipale

**Affiliations:** 1 University of Eastern Finland, Department of Forensic Psychiatry, Niuvanniemi Hospital, Kuopio, Finland; 2 Department of Clinical Neuroscience, Karolinska Institutet, Stockholm, Sweden; 3 Institute for Molecular Medicine Finland, University of Helsinki, Helsinki, Finland; 4 Center for Psychiatry Research, Stockholm City Council, Stockholm, Sweden; 5 Department of Medicine, Solna, Karolinska Institutet, Stockholm, Sweden; 6 Department of Psychiatry and Psychotherapy, School of Medicine, Technical University of Munich, Munich, Germany; 7 The Zucker Hillside Hospital, Department of Psychiatry, Northwell Health, Glen Oaks, USA; 8 Donald and Barbara Zucker School of Medicine at Hofstra/Northwell, Department of Psychiatry and Molecular Medicine, Hempstead, USA; 9 Charité - Universitätsmedizin Berlin, Department of Child and Adolescent Psychiatry, Berlin, Germany; 10 German Center for Mental Health (DZPG), partner site, Berlin, Germany; 11 Department of Clinical Neuroscience, Division of Insurance Medicine, Karolinska Institutet, Stockholm, Sweden; 12 University of Eastern Finland, School of Pharmacy, Kuopio, Finland

**Keywords:** Bipolar disorder, Psychology, Prognostic markers

## Abstract

There are no established prognostic tools for predicting relapse risk in first-episode bipolar disorder (FEBD), limiting the use of personalized treatment approaches. We aimed to develop and validate a machine learning (ML) model to predict relapse in FEBD patients and assess whether pharmacotherapy effectiveness varies across predicted risk strata. We used nationwide registry data from Sweden (n = 30,402; follow-up = 2006–2021) and Finland (n = 13,790; follow-up = 1996–2018). The developed ML model achieved an area under the receiver operating characteristic curve (AUROC) of 0.71 (95% CI = 0.69–0.72, highest for relapse due to psychotic mania [AUROC = 0.85, 95% CI = 0.80–0.89]) in the Swedish cohort (internal validation) and 0.68 (95% CI = 0.66–0.69, highest for nonpsychotic mania [AUROC = 0.74, 95% CI = 0.69–0.78]) in the Finnish cohort (external validation). The model incorporated only seven accessible predictors, including pharmacotherapies during the first 30 days post-FEBD (lithium, combination treatments, and antipsychotics), outpatient follow-up within 30 days post-FEBD, prolonged initial hospitalization for FEBD, history of prior psychiatric hospitalizations, and sickness absence days. Among high-risk patients (N = 10,119, 31.77%), long-acting injectable (LAI) antipsychotics were associated with the greatest reduction in psychiatric rehospitalization risk (HR = 0.44, 95% CI 0.29–0.67). In the low-risk group (n = 21,736, 68.23%), only combinations of quetiapine with valproate (HR = 0.79, 95% CI = 0.65–0.95) or lamotrigine (HR = 0.86, 95% CI = 0.74–0.99) were associated with reduced rehospitalization risk. Available online for research purposes, our internally and externally validated prognostic model may inform clinical decision-making by identifying high-risk individuals who could benefit from early initiation of LAI antipsychotics, promoting a shift toward proactive, risk-guided treatment in FEBD patients.

## Introduction

Most individuals with first-episode bipolar disorder (FEBD) experience relapse during the course of their illness, with annual relapse rates estimated at 21.9−26.3% [1], and nearly half require psychiatric rehospitalization shortly after diagnosis [2]. While epidemiological studies have identified risk factors such as residual symptoms of mania or depression, previous affective relapses, poor baseline functioning, and substance use [3–5], these have not been translated into prognostic tools. In other medical fields, models such as the Framingham risk score routinely guide risk stratification and treatment [6]; however, similar advancements in bipolar disorder (BD) treatment are lacking. Existing efforts to develop prediction models for bipolar relapse have largely focused on advanced illness stages, relying on small (<1000 patients) potentially selected samples without external validation [7–11]. The development of robust prognostic models requires the use of large, representative cohorts with long-term follow-up [12] and crucial external validation to ensure generalizability [13].

Although clinical guidelines emphasize the need to tailor treatment for people with BD to meet individual patient characteristics, their recommendations remain broad [14–16]. This difference in prognostic capability likely contributes to the use of reactive, trial-and-error treatment strategies for BD patients. In some patients, medication regimens become progressively complex over time due to limited responses to initial therapies, often requiring multiple medication classes in the later stages of BD [17]. Additionally, while extensive observational data have suggested that long-acting injectable (LAI) antipsychotics are associated with the lowest risk of rehospitalization for BD [18], their universal applicability in FEBD remains unclear. Overall, it is unclear whether different pharmacotherapies could be effectively targeted through proactive, risk-based strategies during the early stages of BD.

Here, we aimed to develop and validate a novel machine learning (ML) model for predicting relapse risk in patients with FEBD, focusing on creating a parsimonious prognostic model with few routinely collected variables to enhance clinical applicability. Additionally, we examined whether the model could help differentiate the relative effectiveness of pharmacotherapies in reducing psychiatric rehospitalization risk across patients in different risk strata. Finally, given the inherent uncertainty of psychiatric diagnostic boundaries [19], we assessed the model’s transdiagnostic applicability to ensure its usefulness in uncertain cases of BD diagnosis. For these purposes, we used two unselected nationwide cohorts with up to 23 years of follow-up for model development and internal and external validation.

## Methods

### Study design and data acquisition

We followed the TRIPOD + AI reporting guidelines [20]. A prestudy protocol or registration was not prepared, and the study had no public involvement. We analyzed two nationwide registry-based cohorts from Sweden and Finland extracted from comparable registry databases using identical exclusion criteria (details in the Supplementary Methods and Supplementary Fig. 1). Ethical approval was granted by the Regional Ethics Board of Stockholm (decision number: 2007/762-31), the Swedish Ethical Review Authority (2024-08708-02), the Finnish National Institute for Health and Welfare, the Social Insurance Institution of Finland, the Finnish Centre for Pensions, and Statistics Finland (permissions THL/5279/14.06.00/2023, 31/522/2019, 19023, and TK-53-569-19). Given that this study was based on registry data and did not involve direct participant contact, informed consent was not required under the legislation of either country. The study was conducted in accordance with the declaration of Helsinki.

Both cohorts included individuals diagnosed with FEBD (ICD-10: F30-F31) in inpatient or outpatient settings, with a minimum follow-up of 2 years and an age limit of 45 years at inclusion. The Swedish cohort included patients with documented treatment contacts across Sweden followed from July 1, 2006, to December 31, 2021. These patients were identified through the National Patient Register (covering inpatient and specialized outpatient care) and the MiDAS Register (tracking disability pensions and sickness absences). Similarly, the Finnish cohort included individuals diagnosed with FEBD between January 1, 1996, and December 31, 2018. The data for this cohort were sourced from the Hospital Discharge Register maintained by the National Institute of Health and Welfare and sickness absence and disability pension records from the Social Insurance Institution of Finland and the Finnish Centre for Pensions.

For transdiagnostic assessments, we included two additional cohorts from the same Swedish registry databases as the FEBD cohort—individuals with nonaffective first-episode psychosis (FEP; F20–F29) and first-episode psychotic depression (FEPD; F32.3, F33.3)—followed over identical periods.

### Model development and validation

Data analyses were conducted between January 2023 and January 2025. We conducted ML analyses on relapse risk (defined as psychiatric hospitalization due to bipolar relapse) prediction in R, version 4.1.1 (R Project for Statistical Computing). The primary outcome was the prediction performance of all-cause hospitalization due to bipolar relapse (ICD-10: F30-F31) within two years post-FEBD; the secondary outcome was the prediction performance of specific hospitalizations due to BD. The primary ML task was binary classification of 2-year relapse (psychiatric hospitalization due to bipolar relapse vs. no hospitalization), with secondary analyses extending this framework to cause-specific hospitalizations (e.g., psychiatric hospitalization due to mania vs. no hospitalization). Performance was assessed in terms of discrimination using the area under the receiver operating characteristic curve (AUROC) and calibration (i.e., the alignment between predicted and observed probabilities) through calibration plots and metrics (Brier score, calibration slope, and intercept/calibration-in-the-large). We also evaluated the fairness of the predictions by analyzing discrimination and calibration across different subgroups, including immigration status, sex, and educational levels (details in the Supplementary Methods). The potential clinical utility of the model was gauged via decision curve analysis [21] across relapse risk thresholds of 0–40%, as higher thresholds are unlikely to be clinically acceptable (details in the Supplementary Methods). To evaluate the model’s ability to predict long-term outcomes, we used Cox proportional hazards regression to analyze relapse risk across all available follow-up periods and calculated the C-index as a measure of predictive performance.

In the Swedish cohort, the nationwide sample was randomly geographically split into development (10 counties) and validation (the remaining 11 counties) datasets, with the development sample size determined to meet minimum requirements for robust model training. The final model was restricted to a maximum of 15 predictors. A rate of hospitalization due to BD relapse of 6% was assumed based on prior observational data [4], with an anticipated AUROC of 0.70. Minimum sample size calculations, adapted from methods for multivariable models using the *pmsampsize* package [22], yielded a requirement of 4455 individuals with 268 events, ensuring 17.82 events per predictor parameter. Given that machine learning models often require larger samples than regression-based models [23], we doubled this estimate, setting a minimum development sample size of 8910 individuals.

We developed an ML model to predict the risk of hospitalization due to BD within two years following FEBD diagnosis, excluding admissions within 30 days, which were considered part of the initial hospitalization if diagnosed in an inpatient setting. We incorporated a broad set of clinical, sociodemographic, and socioeconomic variables from all available registries at FEBD diagnosis or within the preceding 1–2 years. A total of 79 variables covering clinical history, first-line FEBD treatments, prior medication use, employment history, disability pensions, sickness absences, and demographic factors were explored (details in Supplementary Table 2). First-line treatments were included as predictors since these decisions likely influence the subsequent course of illness. As the actual patient records were unavailable, these predictors were approximated using register-based data. Specifically, treatments (i.e., first pharmacotherapy choices and early outpatient care) initiated within 30 days were used as proxy measures of first-line treatment decisions. Note that there was no temporal overlap between the predictors and the outcome variable, as only the relapses that occurred after 30 days post-FEBD were considered outcomes.

Initial ML modeling was performed using eXtreme Gradient Boosting (XGBoost) [24] within a nested cross-validation framework in the development cohort, incorporating all 79 predictors (details in the Supplementary Methods and Supplementary Fig. 2). Feature selection was then applied, reducing the variable set to the 15 most important predictors based on feature gain. A sequential forward selection (SFS) approach within cross-validation was used to refine the model further, iteratively adding variables until no additional predictors improved performance. The final model, trained on this optimized set, was recalibrated using logistic regression. The contributions of the model’s predictors were estimated using the Shapley Additive Explanation (SHAP) [25]. Performance, assessed through discrimination and calibration, was validated internally on held-out Swedish data, externally on the Finnish cohort, and transdiagnostically in Swedish samples with FEP and FEPD. To evaluate the impact of the modeling strategy, elastic net regression, support vector machine, and random forest models were trained and compared against the final XGBoost model (details in the Supplementary Methods).

### Pharmacoepidemiologic analysis

We investigated whether the association between pharmacotherapy and the risk of all-cause psychiatric hospitalization (ICD-10: all F diagnoses) varied according to the ML model’s predicted risk strata over the total follow-up period (up to 23 years in the Finnish cohort and 15 years in the Swedish cohort). We used all-cause psychiatric hospitalizations to increase event rates, also recognizing that hospitalizations due to other conditions (e.g., suicidal behavior) are partly driven by BD-related behavior in FEBD [26]. The analysis aimed to assess whether the relationship between specific pharmacotherapies, including combination treatments, and psychiatric hospitalization differed across predicted risk levels. The Swedish and Finnish individuals in the validation cohort were stratified using the model’s prediction cutoff, determined by Youden’s index [27], in the development cohort.

The primary treatment exposures included specific mood stabilizers (Anatomical Therapeutic Chemical [ATC] codes N03AF01, N03AG01, N03AX09, and N05AN01), antipsychotics (N05A, excluding lithium), and their combinations, with nonuse of either class serving as the reference group. Medications and their combinations were included if at least 15 outcome events were observed. Medication exposure periods were derived using the PRE2DUP method [28], which estimates time-varying drug use by analyzing prescription purchases, incorporating defined daily doses, purchase amounts, individual use patterns, hospitalization periods, and stockpiling.

Within-individual Cox regression analyses were conducted to examine the association between pharmacotherapy use and psychiatric rehospitalization using SAS version 9.4. The within-individual approach mitigates selection bias by comparing treatment periods within the same individual while accounting for repeated outcome events [29]. The follow-up time was reset to zero after each rehospitalization, and the models were adjusted for temporal factors, including treatment sequence, time since cohort entry, and concomitant psychotropic medication use (e.g., antidepressants, benzodiazepines, ADHD medications, and medications for substance use disorders).

The results from the Swedish and Finnish cohorts were pooled using fixed-effect meta-analysis with the *metafor* package in R [30], generating pooled hazard ratios (HRs) and 95% confidence intervals (CIs) for each pharmacotherapy.

## Results

### Demographic results

We gathered data from 30,402 patients with FEBD from the Swedish cohort (mean [SD] age, 29.68 [8.10] years; 10,085 [33.17%] men; 2-year BD relapse-related hospitalization rate, 2786 [9.16%]); mean [SD] follow-up, 8.53 [3.69] years; individuals on disability pensions at baseline, 2236 [7.35%]; and 13,790 patients from the Finnish cohort (mean [SD] age, 30.73 [8.16] years; 5817 [42.18%] men; 2-year BD relapse-related hospitalization rate, 1949 [14.13%]; mean [SD] follow-up, 10.58 [4.95] years; individuals on disability pensions at baseline, 1240 [8.99%]). In both cohorts (Table 1), bipolar depression was the leading specific cause of rehospitalization, followed by bipolar mania. The Swedish cohort was geographically split, with half of the counties allocated for model development (n = 12,337, exceeding the minimum required sample of 8910) and half for internal validation (n = 18,065). Patients who relapsed within two years post-FEBD were more often diagnosed in inpatient settings; had higher rates of substance use disorder; and were more likely to receive first-line treatment with antidepressants, antipsychotics, mood stabilizers, or benzodiazepines. The transdiagnostic validation samples included 23,362 patients with FEP and 5491 with FEPD (Supplementary Table 1).

**Table 1:** Clinical and Sociodemographic Characteristics of the Study.

Characteristic	Swedish Development Sample (N = 12,337)			Swedish Internal Validation Sample (N = 18,065)			Finnish External Validation Sample (N = 13,790)		
	No relapse within 2 years (N = 11,193)	Relapse within 2 years (N = 1144)	Statistical Testing (T/χ², p-value)	No relapse within 2 years (N = 16,423)	Relapse within 2 years (N = 1642)	Statistical Testing (T/χ², p-value)	No relapse within 2 years (N = 11,841)	Relapse within 2 years (N = 1949)	Statistical Testing (T/χ², p-value)
Age, mean (SD)	29.39 (8.16)	28.62 (8.24)	T = 3.01, p = 0.0027	29.99 (8.02)	29.41 (8.18)	T = 2.75, p = 0.0061	30.73 (8.13)	30.72 (8.32)	T = 0.022, p = 0.9821
Males, N (%)	3599 (32.15)	405 (35.40)	χ² = 4.85, p = 0.0277	5521 (33.62)	560 (34.10)	χ² = 0.14, p = 0.7106	4931 (41.64)	886 (45.46)	χ² = 9.84, p = 0.0017
Any Employment (year before FEBD), N (%)	5984 (53.46)	625 (54.63)	χ² = 0.53, p = 0.4683	9442 (57.49)	888 (54.08)	χ² = 6.96, p = 0.0083	7862 (66.40)	1251 (64.19)	χ² = 3.55, p = 0.0596
Disability Pension at Baseline, N (%)	903 (8.07)	61 (5.33)	χ² = 10.41, p = 0.0013	1181 (7.19)	91 (5.54)	χ² = 5.95, p = 0.0147	1067 (9.01)	173 (8.88)	χ² = 0.02, p = 0.8808
Place of FEBD Diagnosis, N (%)			χ² = 314.19, p < 0.0001			χ² = 606.86, p < 0.0001			χ² = 615.86, p < 0.0001
Inpatient Care	1086 (9.70)	311 (27.19)		1797 (10.94)	531 (32.34)		2172 (18.34)	847 (43.46)	
Outpatient Care	10,107 (90.30)	833 (72.81)		14,626 (89.06)	1111 (67.66)		9669 (81.66)	1102 (56.54)	
Substance Use Disorder (SUD), N (%)	811 (7.25)	122 (10.66)	χ² = 16.87, p = 0.00004	1295 (7.89)	203 (12.36)	χ² = 38.77, p < 0.0001	622 (5.25)	207 (10.62)	χ² = 84.40, p < 0.0001
First Medications Post-FEBD, N (%)									
Any Antipsychotics	2425 (21.67)	498 (43.53)	χ² = 273.28, p < 0.0001	3885 (23.66)	706 (43.00)	χ² = 293.55, p < 0.0001	2591 (21.88)	650 (33.35)	χ² = 121.8, p < 0.0001
Any Mood Stabilizers	5349 (47.79)	635 (55.51)	χ² = 24.45, p < 0.0001	7800 (47.49)	971 (59.14)	χ² = 80.52, p < 0.0001	2677 (22.61)	724 (37.15)	χ² = 189.62, p < 0.0001
Any Antipsychotic + Mood Stabilizer	1041 (9.30)	264 (23.08)	χ² = 206.80, p < 0.0001	1655 (10.08)	407 (24.79)	χ² = 317.97, p < 0.0001	814 (6.87)	282 (14.47)	χ² = 130.9, p < 0.0001
Any Antidepressants	6407 (57.24)	733 (64.07)	χ² = 19.59, p = 0.00001	8806 (53.62)	973 (59.26)	χ² = 18.88, p = 0.00001	5583 (47.15)	947 (48.59)	χ² = 1.33, p = 0.2482
Any Benzodiazepine or Related	1329 (11.87)	239 (20.89)	χ² = 75.27, p < 0.0001	2250 (13.70)	367 (22.35)	χ² = 89.48, p < 0.0001	1289 (10.89)	332 (17.03)	χ² = 60.40, p < 0.0001
Cause of Rehospitalization (ICD-10 codes):									
All Bipolar Depressions (F31.3-F31.5)		374 (32.69)			502 (30.57)			645 (33.09)	
All Bipolar Manias (F30, F31.0-F31.2)		178 (15.56)			270 (16.44)			473 (24.27)	
Non-Psychotic Depression (F31.4)		143 (12.5)			157 (9.56)			332 (17.03)	
Psychotic Depression (F31.5)		18 (1.57)			23 (1.4)			72 (3.69)	
Mixed Episode (F31.6)		78 (6.82)			89 (5.42)			248 (12.72)	
Non-Psychotic Mania (F30.1, F31.1)		51 (4.46)			68 (4.14)			114 (5.85)	
Psychotic Mania (F30.2, F31.2)		71 (6.21)			89 (5.42)			230 (11.8)	
Other and Unspecified (F31.7-F31.9)		514 (44.93)			781 (47.56)			583 (29.91)	

### Model development and validation results

**Fig. 1:**
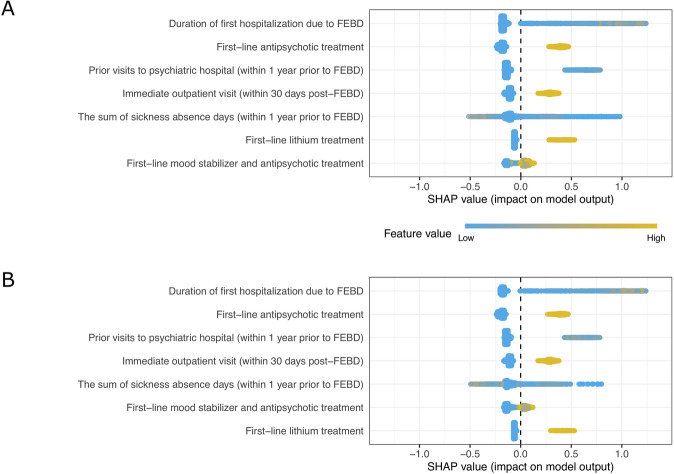
SHAP summary plot illustrating the contributions of individual predictors to the ML model’s relapse risk predictions. Each point represents an observation from a given validation sample for a given predictor (feature). The x-axis displays the SHAP value, which indicates both the direction and magnitude of the predictor’s impact on relapse risk (positive values denote increased risk, negative values denote decreased risk). The color gradient reflects the predictor’s relative value (warm tones for higher, cool for lower), and predictors are ranked from top to bottom by the mean absolute SHAP value. **A** The Swedish internal validation sample and (**B**) the Finnish external validation sample.

In the development cohort, the out-of-training predictions using all 79 predictors within a nested cross-validation framework yielded an AUROC of 0.70 (95% CI = 0.69–0.72). SFS identified seven key predictors of relapse: prolonged first hospitalization for BD, first-line (i.e., the first 30 days post-FEBD) pharmacotherapies (lithium, combination treatments, and antipsychotics), number of psychiatric hospital visits (within one year pre-FEBD), outpatient visit within 30 days post-FEBD, and a high number of previous sickness absence days (Fig. 1).

**Fig. 2:**
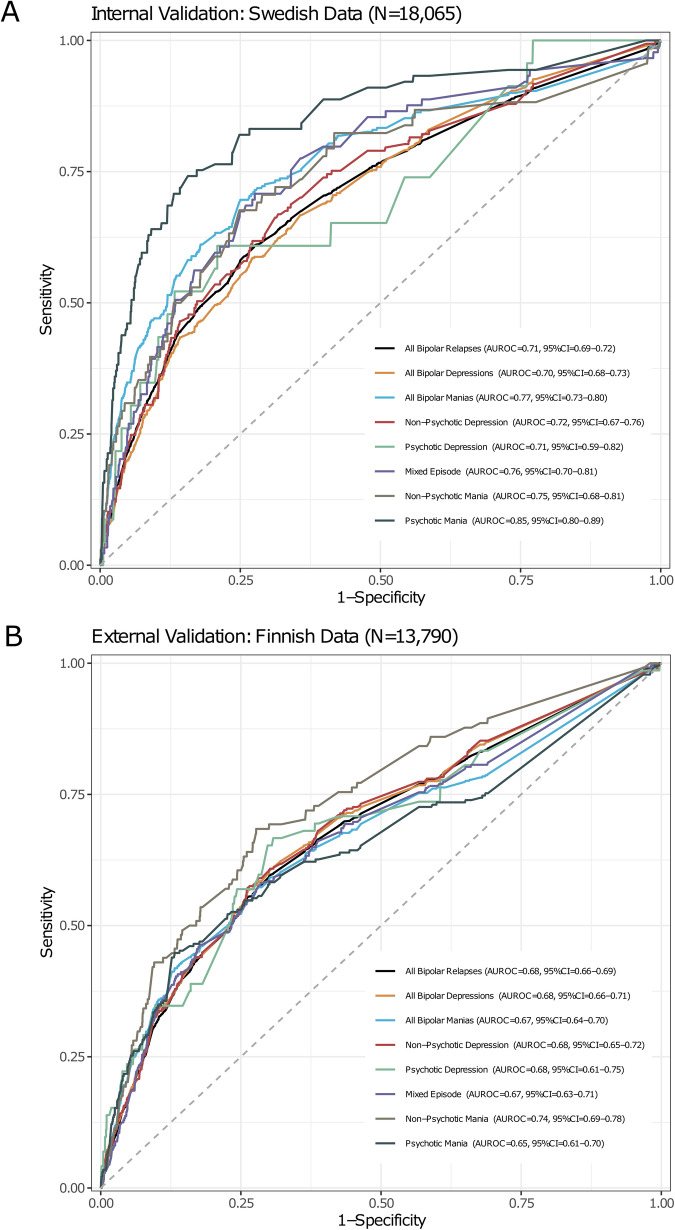
Discrimination ability of the developed prediction model for all-cause bipolar relapse and specific bipolar relapses. **A** The Swedish internal validation sample and (**B**) the Finnish external validation sample.

The final ML model, which included these seven predictors, achieved an AUROC of 0.71 (95% CI = 0.69–0.72) for all-cause relapse within two years in the Swedish cohort (internal validation, Fig. 2), outperforming alternative ML models (Supplementary Fig. 3). The predictive performance was the highest for relapse due to psychotic mania (AUROC = 0.85, 95% CI = 0.80–0.89) and the lowest for psychotic depression (AUROC = 0.71, 95% CI = 0.59–0.82). In the Finnish cohort (external validation; Fig. 2), the model yielded an AUROC of 0.68 (95% CI = 0.66–0.69) for all-cause bipolar relapse, with the highest performance for nonpsychotic mania (AUROC = 0.74, 95% CI = 0.69–0.78) and the lowest for psychotic mania (AUROC = 0.65, 95% CI = 0.61–0.70). An online version of the model with interpretable predictions is available for research purposes: https://johannes-lieslehto.shinyapps.io/biporacle/.

In the development cohort, the optimal cutoff for the ML model, determined using Youden’s index, was 9.15% (details in Supplementary Fig. 4). At this threshold, the model achieved a sensitivity of 61.82%, specificity of 70.83%, positive predictive value (PPV) of 17.49%, and negative predictive value (NPV) of 94.89% in the internal validation sample. In the external Finnish validation sample, the sensitivity was 56.08%, the specificity was 72.80%, the PPV was 25.34%, and the NPV was 90.97%. The detailed discrimination metrics across various thresholds are detailed in Supplementary Tables 3–4.

**Fig. 3:**
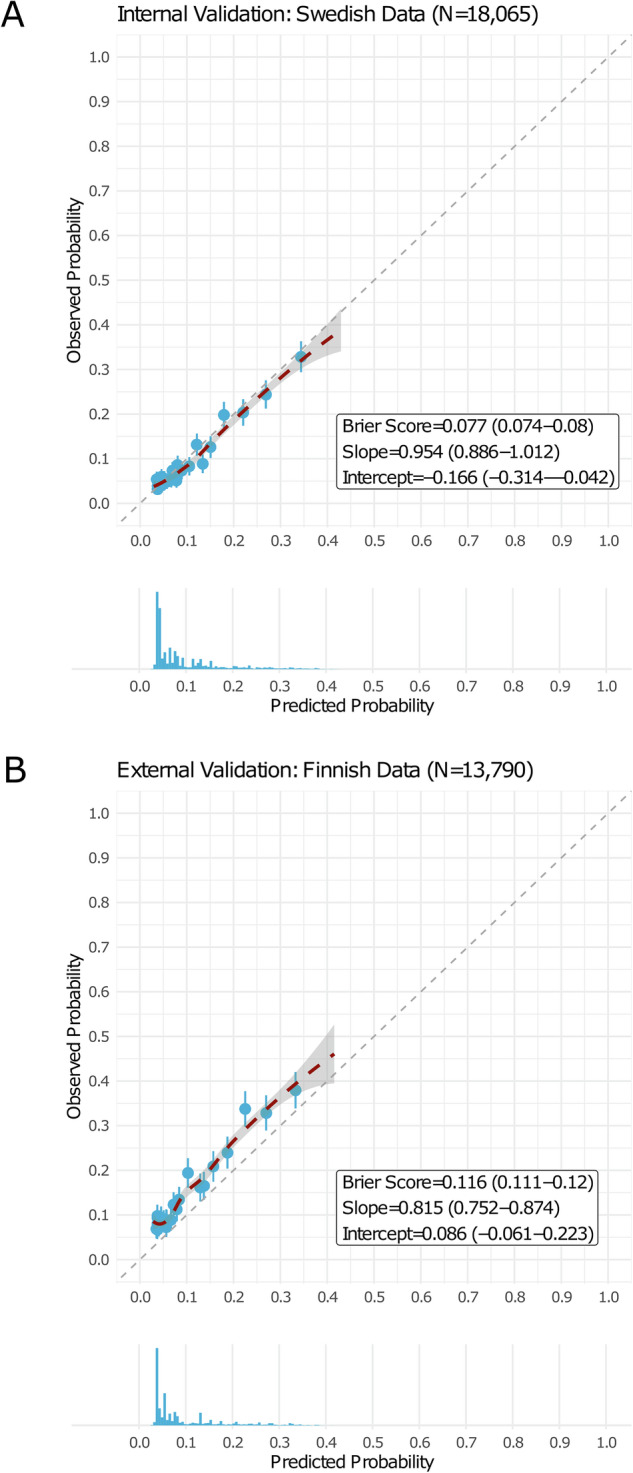
Calibration of the developed prediction model for predicting relapse. **A** The Swedish internal validation sample and (**B**) the Finnish external validation sample. The red dashed lines represent smoothed nonlinear curves generated using a loess smoother with 95% CIs (shaded gray). Histograms displaying the frequency of the model’s probability predictions.

Visual inspection indicated good calibration in the internal validation sample but slight underestimation in the external validation sample (Fig. 3). In the internal validation, the Brier score was 0.08 (95% CI = 0.074–0.08), with a calibration slope of 0.95 (95% CI = 0.89–1.01) and an intercept of −0.17 (95% CI = − 0.31 to −0.04). According to the external validation, the Brier score was 0.12 (95% CI = 0.11–0.12), with a calibration slope of 0.82 (95% CI = 0.75–0.87) and an intercept of 0.09 (95% CI = − 0.06–0.22). No significant prediction bias was detected for immigration status, sex, or education level (all P > 0.1; Supplementary Table 5). Decision curve analysis suggested potential clinical benefits across relapse risk thresholds of 4–34% in the internal validation cohort and 9–40% in the external validation cohort (Supplementary Fig. 5 in the Supplement).

We also conducted time-to-event analyses using the available follow-up data (Supplementary Figs. 6, 7). In the Swedish internal validation cohort, the 15-year relapse rates among patients in the top 20% of the predicted risk quintiles were 45.57% (95% CI, 41.91–49.01%) and 13.16% (95% CI, 11.49–14.79%) for those within the bottom 20% of the risk-predicted relapse (HR, 4.61 [95% CI, 4.12-5.16]; Harrell’s C-index of the developed model = 0.67 [95% CI, 0.66-0.68]). In the Finnish validation cohort, the 23-year relapse rate was 46.6% (95% CI, 44.23−48.87%) for patients within the top 20% of the total risk of relapse and 15.98% (95% CI, 13.82−18.08%) for those predicted to survive (HR, 3.84 [95% CI, 3.46–4.26]; Harrell’s C-index of the developed model = 0.65 [95% CI, 0.64–0.66]).

In the transdiagnostic assessment, the model achieved an AUROC of 0.68 (95% CI = 0.65–0.71) for predicting relapse in FEPD patients and 0.60 (95% CI = 0.59–0.61) in FEP patients (Supplementary Fig. 8). Calibration metrics and visual inspection indicated poor calibration for both disorders (Supplementary Fig. 9).

### Pharmacoepidemiologic results

**Fig. 4:**
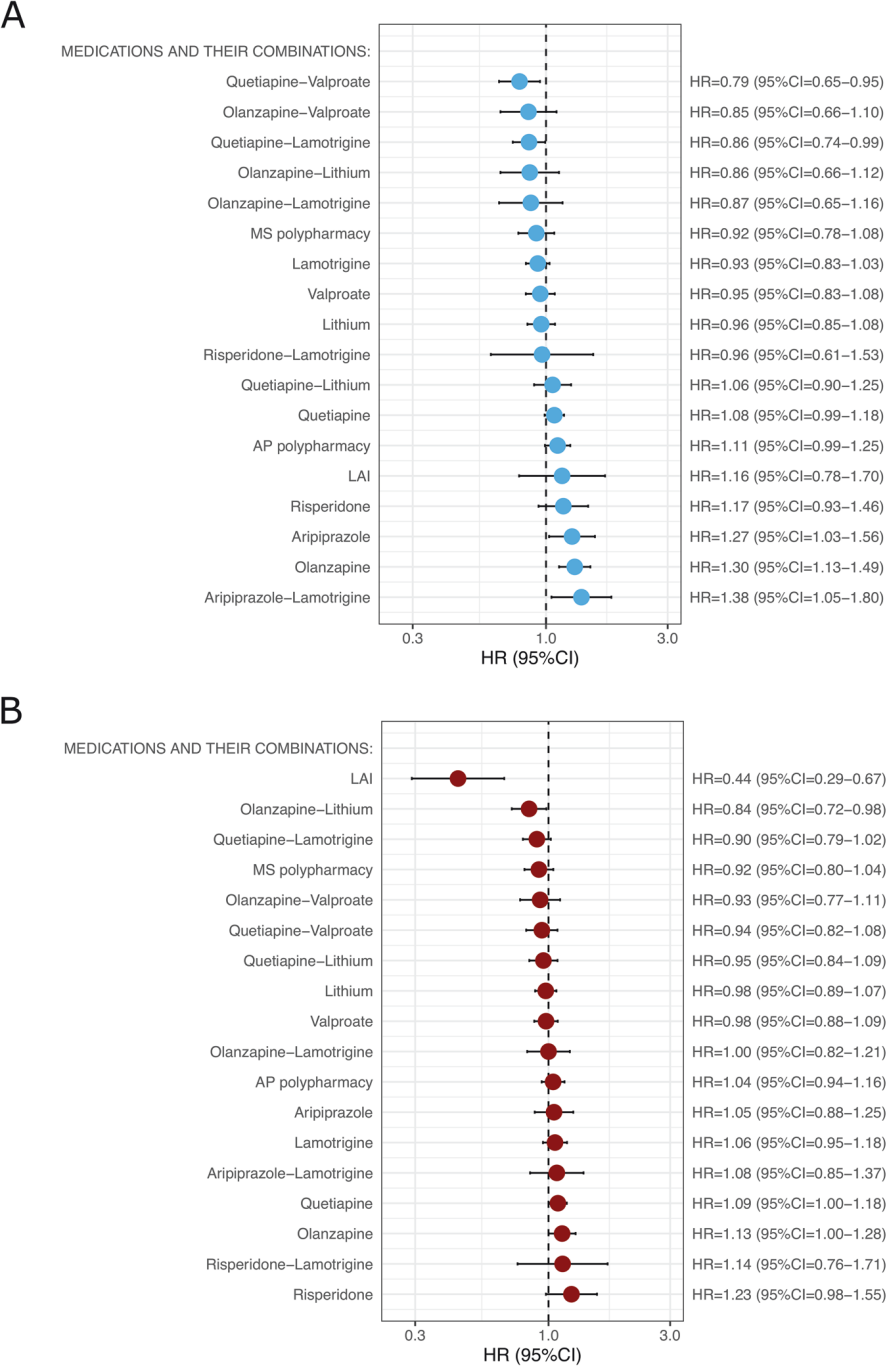
Within-individual pharmacoepidemiologic meta-analysis of the associations of pharmacotherapy with psychiatric rehospitalization risk in both cohorts. **A** Individuals with a predicted low relapse risk (68.23%, N = 21,736) and (**B**) individuals with a predicted high relapse risk (31.77%, N = 10,119).

Using Youden’s index (threshold 9.15%), patients in the two validation cohorts were classified into a low-risk subgroup (<9.15% predicted relapse risk, 68.23%, N = 21,736) and a high-risk subgroup ( ≥ 9.15% predicted relapse risk, 31.77%, N = 10,119). In the low-risk group (Fig. 4A), the combination of quetiapine and valproate was associated with the lowest adjusted hazard ratio (HR) for psychiatric rehospitalization (HR = 0.79, 95% CI = 0.65–0.95). Furthermore, the periods of use of quetiapine and lamotrigine (vs. nonuse periods of antipsychotics or mood stabilizers) were associated with a reduced risk of psychiatric rehospitalization (HR = 0.86, 95% CI = 0.74 − 0.99). Among high-risk patients (Fig. 4B), LAI antipsychotic treatment was associated with the lowest risk of future psychiatric rehospitalization compared with the same individuals’ nonuse periods of antipsychotics or mood stabilizers (HR = 0.44, 95% CI = 0.29–0.67). In the low-risk group, the same was not true (HR = 1.16, 95% CI = 0.78–1.70). In addition to LAIs, the combination of olanzapine and lithium was related to a lower risk of psychiatric rehospitalization among high-risk patients (HR = 0.84, 95% CI = 0.72 − 0.98).

## Discussion

Using two nationwide patient cohorts, we developed an interpretable web-based ML model incorporating seven routinely measured clinical variables that predict future relapse in FEBD patients with performance comparable to that of established risk calculators, such as the Framingham risk score [6]. The developed model with only a handful of predictors was developed to enhance parsimony and improve feasibility for clinical implementation. The model exhibited generalizable predictive performance without bias toward immigration status, sex, or education level. Decision curve analyses supported the model’s potential clinical utility across relevant risk thresholds. Pharmacoepidemiologic findings indicated that the effectiveness of different pharmacotherapies differs according to patients’ predicted risk of relapse. Although the present model is not sufficient for direct clinical implementation, it represents an important proof-of-concept demonstrating the feasibility of developing the first externally validated relapse risk prediction model for FEBD patients.

Previous research has indicated that unaided clinicians overestimate favorable outcomes for low-frequency events such as suicidal behavior [31], underscoring the need for predictive models to enhance risk assessment. The internally and externally validated prediction model represents a step forward in developing individualized risk assessment tools for BD. The model incorporated a small number of key predictors consistent with prior epidemiological and previous ML research on predicting bipolar relapse, including first-line pharmacotherapy (lithium, combination treatments, antipsychotics), immediate outpatient follow-up post-FEBD, and prolonged first hospitalization. These predictors likely reflect baseline illness severity, with more intensive pharmacotherapy and longer hospitalizations marking greater symptom burden at FEBD and associated treatment complexity, consistent with previous research [3, 4, 7–10]. The association between prior psychiatric hospitalizations and relapse risk also aligns with established evidence [5, 11].

Risk prediction enables stratified care to allocate intensive interventions to those most likely to benefit while sparing unnecessary interventions among lower-risk individuals. However, unclear diagnostic boundaries—for instance, conversion rates between schizophrenia and bipolar disorder of 4.5–10.1% [32] and up to 17% of depression cases later reclassified as bipolar disorder [33]—challenge the development of a psychiatric prediction model. To assess the applicability of our model in diagnostically uncertain patients, we evaluated its transdiagnostic performance, which demonstrated its predictive value for FEPD and FEP. While adaptable, its use beyond FEBD will require recalibration and potentially the addition of disorder-specific variables. Nonetheless, the observed converging performance indicates shared relapse mechanisms across FEBD, FEP, and FEPD patients.

We observed variations in relapse risk across different pharmacotherapies via an individual’s predicted risk profile. Among high-risk patients (approximately one-third of whom are at risk), LAI antipsychotics were associated with the greatest reduction in psychiatric rehospitalization risk, potentially reflecting their efficacy in managing mania and addressing treatment nonadherence [34, 35]. These findings also align with the model’s strongest predictive performance for mania-related relapses. In contrast, LAI antipsychotic use was not linked to reduced psychiatric rehospitalization risk in the majority of patients predicted to have a low risk of relapse. Interestingly, among these individuals, only quetiapine in combination with lamotrigine or valproate was linked to a decreased risk of psychiatric rehospitalization. Given the established efficacy of these combinations in treating depressive symptoms [36, 37], it is plausible that lower-risk patients predominantly exhibit depressive polarity.

The pharmacoepidemiologic findings across risk groups highlight the limitations of a one-size-fits-all approach and demonstrate the potential of ML to guide personalized treatment in BD patients, similar to stratified treatment strategies routinely employed in other medical fields (e.g., oncology [38]). Our results suggest that an individual’s risk profile may influence the effectiveness of specific pharmacotherapies. Although LAI antipsychotics are widely recognized as effective at preventing psychiatric hospitalizations in patients with BD [18, 34], our findings indicate that this benefit may be primarily driven by a subgroup of high-risk patients, with the majority of patients deriving less pronounced benefits. If prospectively validated, these results could inform treatment guidelines that emphasize risk-based pharmacotherapy selection at baseline, moving away from the current reactive paradigm, where new therapies are typically introduced after treatment failure [17, 39].

### Strengths and limitations

This study’s strengths include the use of two large, unselected nationwide cohorts with up to two decades of follow-up. This approach enabled the development and internal and external validation of an ML-based risk prediction model in more than 40,000 patients with FEBD, enhancing its generalizability. The within-individual design of the pharmacoepidemiologic analysis mitigates selection bias by using patients as their own controls, a key limitation in observational studies [29]. Nonetheless, the model’s real-world clinical utility remains unproven in the absence of clinician-derived relapse risk benchmarks and, given its dependence on the availability of all seven predictors, which may not be consistent in routine practice. Additionally, observational data preclude causal inference, and despite within-individual analysis, confounding by indication cannot be excluded in pharmacoepidemiologic analyses. Prospective trials are therefore needed to evaluate the clinical impact of the model on treatment selection. Additionally, nonpsychotic major depressive episodes, which are common initial presentations of bipolar disorder, could not be included as index episodes because they are infrequently treated in hospital settings; thus, their outcomes are not reliably captured in registers.

## Conclusions

This prognostic study developed and externally validated a relapse risk model using routine clinical data. The prediction model, available online for research purposes, may facilitate the identification of high-risk individuals and inform more tailored pharmacotherapy in FEBD patients.

## Supplementary information

Supplementary Material 1

## Data Availability

The data used in this study cannot be made publicly available due to privacy regulations. According to the General Data Protection Regulation, the Swedish law SFS 2018:218, the Swedish Data Protection Act, the Swedish Ethical Review Act, and the Public Access to Information and Secrecy Act, these types of sensitive data can be made available only for specific purposes, including research, that meets the criteria for accessing sensitive and confidential data as determined by a legal review. The readers may contact Professor Kristina Alexanderson (kristina.alexanderson@ki.se) regarding the Swedish data. The Finnish data collected for this study are proprietary to Finnish government agencies, the Social Insurance Institution of Finland, and the National Institute for Health and Welfare, which granted the researchers permission and access to the data. The data supporting this study’s findings are available from these authorities, but restrictions apply to the availability of these data.

## Code availability

The analysis codes used to analyze these data are available at https://github.com/johpulkk/FEBD_relapse_ML.
