# Supplementary material for: Relapse risk prediction in patients with first-episode bipolar disorder: development, external validation, and pharmacotherapy associations of a machine learning model
Source: Mol Psychiatry. 2025 Oct 23;30(12):5722–30. doi: 10.1038/s41380-025-03316-2 (PMC12602334; doi:10.1038/s41380-025-03316-2)
Supplement: Supplementary file 1 — Supplementary Material 1 [file 41380_2025_3316_MOESM1_ESM.docx]

**Supplementary Material**

**Relapse Risk Prediction in Patients with First-Episode Bipolar Disorder: Development, External Validation, and Pharmacotherapy Associations of a Machine Learning Model**

**Supplementary Methods**

**Supplementary Tables 1-5**

**Supplementary Figures 1-9**

**Supplementary References**

**Supplementary Methods.**

- 1. **Datasets**

The Swedish cohort was identified using data from the National Patient Register (capturing all inpatient and specialized outpatient care episodes with diagnostic codes from 2006 to 2021) and the MiDAS Register (disability pensions and sickness absences with diagnoses). Other information was additionally collected from the Prescribed Drug Register (prescription drug purchases from July 2005 to December 2021), the Causes of Death Register (cause-of-death data from 2006 to 2021), and the LISA register (providing demographic and socioeconomic information). Individuals with a first-time diagnosis of bipolar disorder (ICD-10: F30–F31) between 2006 and 2021, without any preceding diagnosis of schizophrenia-spectrum disorders (F20–F29), were initially included (N=71,417). To ensure the cohort represented first-episode cases, individuals who used antipsychotics or mood stabilizers (ATC codes: N05A, N03AF01, N03AG01, N03AX09) in three to 15 months preceding the FEBD diagnosis were excluded (N=22,524), resulting in 48,893 participants. Subsequent exclusions were applied for individuals who died during the first hospitalization, had a first hospitalization exceeding 100 days, emigrated during the two-year follow-up, had less than two years of follow-up data, or received a schizophrenia-spectrum disorder (F20–F29) diagnosis during the follow-up period (N=6,542), leaving 42,351 individuals. To maintain a focus on early adulthood onset, participants aged below 15 or above 45 years at the time of diagnosis were excluded (N=11,949). The final Swedish cohort comprised 30,402 individuals, which was randomly geographically divided for model development (10 counties, n=12,337) and internal validation (11 remaining counties, n=18,065).

The Finnish cohort was identified from nationwide registers, including the Hospital Discharge Register (capturing all inpatient care episodes with diagnoses from 1987 to 2018 and specialized outpatient care visits from 1998 to 2018), from sickness absences (from the Social Insurance Institution of Finland) and disability pensions (from the Social Insurance Institution of Finland and the Finnish Centre for Pensions). Other information was additionally collected from the Prescription Register (reimbursed prescription drug purchases from 1995 to 2018), and the Causes of Death Register maintained by Statistics Finland (covering deaths from 1987 to 2018). For the Finnish cohort, we used a previously formed first-episode cohort^1,2^. The primary distinction from the Swedish cohort was the application of a one-year washout period immediately preceding the diagnosis. The initial Finnish cohort included individuals diagnosed with FEBD (ICD-10: F30–F31) between 1996 and 2018, following a one-year washout period for antipsychotics and mood stabilizers, and without preceding schizophrenia-spectrum diagnoses (F20–F29) (N=24,413). Exclusions were applied for those who died during the first hospitalization, had a first hospitalization exceeding 100 days, had less than two years of follow-up based on data linkage, or received a schizophrenia-spectrum disorder diagnosis (F20-F29) within the two-year follow-up period (N=3,333), resulting in 21,080 individuals. Finally, participants aged below 15 or above 45 years at diagnosis were excluded (N=7,290), leaving a final Finnish cohort of 13,790 individuals for external validation.

- 1. **Machine Learning Pipeline of the Model Development**

We developed a machine learning (ML) model to predict the risk of psychiatric rehospitalization due to bipolar disorder (ICD-10: F30–F31) within two years following a first-episode bipolar disorder (FEBD) diagnosis. Rehospitalizations occurring within 30 days of discharge were considered part of the initial hospitalization and excluded from the analysis. The model incorporated several clinical, sociodemographic, and socioeconomic predictors obtained from national registers, reflecting clinical history, first-line pharmacotherapy (initiated within 30 days post-diagnosis), prior medication use, employment history, disability pensions, sickness absences, and demographic characteristics (detailed list of the variables in Supplementary Table 2). As a preprocessing step, we ensured that no predictor variable included in the training had more than 20% missing data, and no participant had more than 40% missing data, resulting in 79 candidate predictors.

The ML modeling was conducted using R, version 4.1.1 (R Project for Statistical Computing). We utilized *caret*^3^ and *mlr*^4^ packages for ML training and validation. The initial ML modeling utilized eXtreme Gradient Boosting (XGBoost)^5^ within a nested cross-validation framework to optimize hyperparameters and prevent overfitting. Missing values were not imputed, as XGBoost inherently handles missing data. The nested cross-validation consisted of a 10-fold outer loop for model validation and an inner loop for hyperparameter tuning. The following hyperparameter search space was explored using grid search optimization using balanced accuracy as a criterion: *eta/learning_rate* (2⁻⁸, 2⁻⁶, 2⁻⁴, 2⁻²), *gamma* (2⁻¹⁶, 2⁻⁶, 2²), *max_depth* (3, 5, 7), *nrounds*/number of decision trees (100, 300, 500), subsampling ratio fixed at 0.5 and *min_child_weight* (2⁻¹⁶, 2⁻⁶, 2⁴, 2⁸). The scale for positive class weighting was determined by class imbalance in the development sample.

After initial modeling in the development sample, feature importance was assessed using the *feature gain* metric from XGBoost. Based on this metric, the top 15 predictors were selected for further analysis. To identify the smallest subset of predictors that maximized performance, a sequential forward selection (SFS) approach was then employed. Limiting the search of predictors to the top 15 variables, this iterative process added predictors one by one, retaining only those that improved balanced accuracy (i.e., mean of sensitivity and specificity). The final selected model was recalibrated using logistic regression to improve probability estimates. The final model’s performance (discrimination and calibration) was then evaluated in the validation samples and transdiagnostic validation samples.

- 1. **Alternative Machine Learning Modeling Strategies**

To benchmark the primary XGBoost model described above, we compared its performance against three alternative commonly used ML algorithms: elastic net regression, support vector machine (SVM), and random forest. As these algorithms do not inherently accommodate missing data, we applied K-nearest neighbor imputation as a preprocessing step. Additionally, all predictors were scaled and standardized to zero.

The elastic net regression model was trained using hyperparameters for lambda (0, 0.2, 0.5, 0.8, 1, 2, 5, 10) and alpha (0, 0.2, 0.4, 0.6, 0.8, 1) that were optimized using grid search using balanced accuracy as an optimization criterion within cross-validation. The linear SVM model was trained using regularization parameters that were optimized within cross-validation: 2⁻⁸, 2⁻⁶, 2⁻⁴, 2⁻², 2⁰, 2², 2⁴, 2⁶, and 2⁸. For the random forest model, the following hyperparameters were optimized: number of trees/*ntree* (500), number of variables tried at each split/*mtry* (6, 8, 10, 12), minimum node size/*nodesize* (1, 3, 5) and maximum number of terminal nodes/*maxnodes* (5, 10, 15).

All alternative ML models were trained using the development sample, and discrimination performance was assessed in the internal validation sample. De Long’s test was employed to compare the area under the receiver operating characteristic curves (AUROC) across these alternative ML models, with the XGBoost model serving as the reference.

- 1. **Clinical Usefulness Comparison to Alternative Assessments**

We conducted a decision curve analysis (DCA)^6^ to assess the potential clinical utility of the developed ML model in predicting bipolar relapse within two years following a first-episode bipolar disorder (FEBD) diagnosis. The DCA quantifies the net benefit of using the model to guide clinical decisions across a range of threshold probabilities, representing the predicted risk at which a clinician might choose to intervene. We assessed the developed model alongside two standard reference strategies: 1) Treat All (assuming that all patients would receive intensive follow-up or interventions), and 2) Treat None (assuming that no patients would receive additional interventions). The DCA was performed separately for the internal validation sample (Swedish, N=18,065) and the external validation sample (Finnish, N=13,790).

- 1. **Fairness Analysis**

We evaluated algorithmic fairness, defined as the absence of bias in model performance across vulnerable subgroups, by examining key performance metrics (AUROC, Brier score, calibration slope, and intercept) stratified by immigration status, gender, and education level (recorded during the year preceding FEBD) within the internal validation dataset. Statistical comparisons between subgroups were conducted by comparing the observed differences in performance (e.g., AUROC variations between males and females) against a null distribution generated through 1,000 random permutations of subgroup labels.

**Supplementary Table 1 Clinical and Sociodemographic Characteristics of the Two Independent Transdiagnostic Samples from Sweden.**

| **Characteristic** | **First-Episode Non-Affective Psychosis (N=23,362)** | |  | **Psychotic Depression (N=5,491)** | |  |
| --- | --- | --- | --- | --- | --- | --- |
|  | \| **No Relapse within 2 years (N=18,318)** \| \| --- \| | \| **Relapse within 2 years (N=5,044)** \| \| --- \| | **Statistical Testing (T/χ², p-value)** | **No Relapse within 2 years (N=5,173)** | **Relapse within 2 years (N=318)** | **Statistical Testing (T/χ², p-value)** |
| Age, mean (SD) | 29.16 (8.20) | 28.43 (7.73) | T=5.86, p<0.0001 | 31.50 (8.72) | 32.61 (8.54) | T=-2.25, p=0.0250 |
| Males, N (%) | 11,179 (61.03) | 3,283 (65.09) | χ²=27.47, p<0.0001 | 2,289 (44.25) | 119 (37.42) | χ²=5.40, p=0.0202 |
| Any Employment (year before FEBD), N (%) | 6,397 (34.92) | 1,549 (30.71) | χ²=31.08, p<0.0001 | 2,480 (47.94) | 161 (50.63) | χ²=0.76, p=0.3825 |
| Disability Pension at Baseline, N (%) | 2,331 (12.73) | 526 (10.43) | χ²=19.23, p<0.0001 | 489 (9.45) | 42 (13.21) | χ²=4.41, p=0.0356 |
| Place of FEBD Diagnosis, N (%) |  |  | χ²=287.70, p<0.0001 |  |  | χ²=76.10, p<0.0001 |
| Inpatient Care | 9,068 (49.50) | 3,177 (62.99) |  | 1,797 (34.74) | 188 (59.12) |  |
| Outpatient Care | 9,250 (50.50) | 1,867 (37.01) |  | 3,376 (65.26) | 130 (40.88) |  |
| Substance Use Disorder (SUD), N (%) | 2,487 (13.58) | 724 (14.35) | χ²=1.95, p=0.1628 | 345 (6.67) | 15 (4.72) | χ²=1.56, p=0.2119 |
| First Medications Post-FEBD, N (%) |  |  |  |  |  |  |
| Any Antipsychotics | 9,819 (53.60) | 2,869 (56.88) | χ²=16.98, p<0.0001 | 2,299 (44.44) | 201 (63.21) | χ²=41.78, p<0.0001 |
| Any Mood Stabilizers | 1,190 (6.50) | 230 (4.56) | χ²=25.64, p<0.0001 | 296 (5.72) | 24 (7.55) | χ²=1.50, p=0.2205 |
| Any Antipsychotic + Mood Stabilizer | 808 (4.41) | 189 (3.75) | χ²=4.11, p=0.0427 | 166 (3.21) | 22 (6.92) | χ²=11.37, p=0.0007 |
| Any Antidepressants | 5,653 (30.86) | 1,078 (21.37) | χ²=173.14, p<0.0001 | 4,112 (79.49) | 264 (83.02) | χ²=2.09, p=0.1480 |
| Any Benzodiazepine or Related | 2,662 (14.53) | 602 (11.93) | χ²=21.98, p<0.0001 | 1,160 (22.42) | 96 (30.19) | χ²=9.80, p=0.0017 |

**Supplementary Table 2. List of All 79 Variables Used for Training the Machine Learning Model to Predict All-Cause Bipolar Relapse Within Two Years After First-Episode Bipolar Disorder (FEBD) Diagnosis, With Corresponding Importance (Gain) Scores in the Development Sample.**

| **Variable Name** | **Importance (Gain)** |
| --- | --- |
| Days of the first inpatient stay for those who received their FEBD diagnosis from inpatient care (zero for others) | 0.168 |
| Treatment with antipsychotics during 30 days after FEBD (yes/no) | 0.103 |
| Total sickness absence days year before FEBD | 0.075 |
| Income from work during a previous calendar year before FEBD (brutto, as kronor) | 0.069 |
| Age at FEBD (years) | 0.061 |
| Whether a person has visited a psychiatric hospital a year before the FEBD | 0.057 |
| Number of all-cause hospitalizations years before FEBD | 0.045 |
| Specialized outpatient visit due to bipolar disorder within 30 days after FEBD (yes vs. No) | 0.045 |
| Combination of Antipsychotic (AP) & Mood Stabilizer (MS) Treatment (0 = no ap or ms treatment, 1 = ap or ms treatment, 2 = ap + ms treatment) 30 days post-FEBD | 0.045 |
| Lithium during 30 days after FEBD | 0.038 |
| Benzodiazepines during 30 days after FEBD | 0.034 |
| Olanzapine treatment during 30 days after FEBD (yes/no) | 0.032 |
| "Z-drug" use during 30 days after FEBD | 0.027 |
| The sum of unemployment days during a previous calendar year before FEBD | 0.022 |
| Antidepressants during 30 days after FEBD (yes/no) | 0.015 |
| Mood stabilizer treatment during 30 days after FEBD | 0.012 |
| severity of major depressive disorder one year before FEBD | 0.011 |
| Education: only elementary level (i.e., 9 years) education (yes/no) | 0.010 |
| Male gender (yes/no) | 0.010 |
| Family situation: Single without children (yes/no) | 0.009 |
| SUD drug use during 30 days after FEBD | 0.009 |
| Parents with non-affective psychosis other than schizophrenia (ICD-10: F21-F24, F26-F29) | 0.009 |
| Education: high-school level (i.e., 10-12 years) education (yes/no) | 0.009 |
| Parental education the highest level of education evaluated by all known parents | 0.007 |
| Ongoing disability pension at the baseline | 0.007 |
| ADHD (ICD-10: F90) comorbidity year before FEBD (yes/no) | 0.007 |
| Depression (ICD-10: F32-F33) comorbidity year before FEBD (yes/no) | 0.007 |
| Parental with alcohol-use disorder | 0.006 |
| Family situation: married or cohabitant with children | 0.006 |
| Suicide attempt (ICD-10: X60-X84, Y10-Y34) year before FEBD | 0.006 |
| Parent with anxiety disorder (yes/no) | 0.006 |
| Born outside of Europe (yes/no) | 0.006 |
| Any employment during a previous calendar year before FEBD (yes/no) | 0.006 |
| Parent with stress-related disorder (ICD-10: F43) (yes/no) | 0.005 |
| Family situation: youth (<=20 years) living at home (with their parents) (yes/no) | 0.005 |
| Parent with depression (yes/no) | 0.005 |
| Born in Sweden (yes/no) | 0.005 |
| Parent with substance use disorder (ICD-10: F11-F19) (yes/no) | 0.005 |
| Other anxiety disorder (ICD-10: F41) comorbidity year before FEBD (yes/no) | 0.004 |
| Anxiety disorder (ICD-10: F40-F43) comorbidity year before FEBD (yes/no) | 0.004 |
| Parent with other anxiety disorders (yes/no) | 0.004 |
| Education: university/college (i.e., >12 years) education (yes/no) | 0.004 |
| Family situation: single with children (yes/no) | 0.004 |
| Parent with bipolar disorder (yes/no) | 0.003 |
| Generalized anxiety disorder (ICD-10: F41.1) comorbidity year before FEBD (yes/no) | 0.003 |
| Phobia (ICD-10: F40) comorbidity year before FEBD (yes/no) | 0.003 |
| Born in Europe (outside of Sweden) (yes/no) | 0.003 |
| Aripiprazole N05AX12 during 30 days after FEBD | 0.003 |
| Mirtazapine during 30 days after FEBD | 0.003 |
| Quetiapine treatment during 30 days after FEBD (yes/no) | 0.002 |
| Stress-related (ICD-10: F43) comorbidity year before FEBD (yes/no) | 0.002 |
| Parent with panic disorder (yes/no) | 0.002 |
| Personality disorder (ICD-10: F60-F69) comorbidity year before FEBD (yes/no) | 0.002 |
| Parent with phobia (yes/no) | 0.002 |
| Eating disorder (ICD-10: F50) comorbidity year before FEBD (yes/no) | 0.002 |
| Parent with schizophrenia (ICD-10: F20 andF25) (yes/no) | 0.002 |
| Any substance use comorbidity (ICD-10: F10-19) year before the FEBD (yes/no) | 0.001 |
| Panic disorder F41.0 | 0.001 |
| Parent with personality disorder (yes/no) | 0.001 |
| Risperidone treatment during 30 days after FEBD (yes/no) | 0.001 |
| Parent with generalized anxiety (yes/no) | 0.001 |
| Parent with ADHD (yes/no) | 0.001 |
| Whether a person had a specialized outpatient visit due to SUD (F1*) within 1 month after ced (yes/no) | 0.001 |
| Parent with suicidal behavior (yes/no) | 0.001 |
| Valproate use during 30 days after FEBD | 0.001 |
| Parent with an eating disorder (yes/no) | 0.001 |
| Parent with PTSD (yes/no) | 0.001 |
| Parent with substance-induced psychosis (ICD-10: F1X.5) | 0.001 |
| Autism (ICD-10: F84) comorbidity year before FEBD (yes/no) | 0.000 |
| Parent with autism (yes/no) | 0.000 |
| Family situation: married or cohabitant without children | 0.000 |
| Parent with OCD (yes/no) | 0.000 |
| Obsessive-compulsive disorder (ICD-10: F42) comorbidity year before FEBD (yes/no) | 0.000 |
| Substance-induced psychosis (F1X.5) year before FEBD | 0.000 |
| Lamotrigine use during 30 days after FEBD | 0.000 |
| Long-acting injectable antipsychotic use during 30 days after FEBD | 0.000 |
| Parent with a developmental disorder | 0.000 |
| Post-traumatic stress disorder (ICD-10: F43.1) comorbidity year before FEBD (yes/no) | 0.000 |
| Developmental disorders (ICD-10: F80-89) comorbidity year before FEBD (yes/no) | 0.000 |

**Supplementary Table 3. Classification Performance of Machine Learning Model Predictions for Two-Year Relapse in Bipolar Disorder Across Varying Threshold Levels in the Swedish Internal Validation Cohort (N=18,065).**

| **Thr%** | **TP (%)** | **TN (%)** | **FP (%)** | **FN (%)** | **Sens%** | **Spec%** | **BAC%** | **PPV%** | **NPV%** | **LR+** | **LR-** | **F1%** | **MCC%** |
| --- | --- | --- | --- | --- | --- | --- | --- | --- | --- | --- | --- | --- | --- |
| 3% | 1948 (14.13) | 18 (0.13) | 11823 (85.74) | 1 (0.01) | 99.88 | 0.09 | 49.98 | 9.09 | 87.50 | 1.00 | 1.43 | 16.66 | -0.35 |
| 4% | 1624 (11.78) | 3737 (27.10) | 8104 (58.77) | 325 (2.36) | 90.38 | 23.33 | 56.85 | 10.54 | 96.04 | 1.18 | 0.41 | 18.88 | 9.50 |
| 5% | 1521 (11.03) | 4743 (34.39) | 7098 (51.47) | 428 (3.10) | 80.09 | 43.62 | 61.85 | 12.44 | 95.63 | 1.42 | 0.46 | 21.53 | 13.83 |
| 6% | 1364 (9.89) | 6580 (47.72) | 5261 (38.15) | 585 (4.24) | 76.67 | 50.16 | 63.42 | 13.33 | 95.56 | 1.54 | 0.47 | 22.71 | 15.44 |
| 7% | 1283 (9.30) | 7301 (52.94) | 4540 (32.92) | 666 (4.83) | 71.74 | 57.86 | 64.80 | 14.55 | 95.34 | 1.70 | 0.49 | 24.19 | 17.11 |
| 8% | 1171 (8.49) | 8174 (59.27) | 3667 (26.59) | 778 (5.64) | 66.08 | 65.93 | 66.00 | 16.24 | 95.11 | 1.94 | 0.51 | 26.08 | 19.06 |
| 9% | 1105 (8.01) | 8559 (62.07) | 3282 (23.80) | 844 (6.12) | 63.34 | 68.70 | 66.02 | 16.83 | 94.93 | 2.02 | 0.53 | 26.59 | 19.41 |
| 10% | 1068 (7.74) | 8762 (63.54) | 3079 (22.33) | 881 (6.39) | 60.60 | 72.54 | 66.57 | 18.08 | 94.85 | 2.21 | 0.54 | 27.85 | 20.70 |
| 15% | 807 (5.85) | 9950 (72.15) | 1891 (13.71) | 1142 (8.28) | 46.41 | 84.23 | 65.32 | 22.73 | 94.02 | 2.94 | 0.64 | 30.52 | 22.65 |
| 20% | 616 (4.47) | 10657 (77.28) | 1184 (8.59) | 1333 (9.67) | 34.71 | 89.81 | 62.26 | 25.40 | 93.22 | 3.41 | 0.73 | 29.34 | 21.37 |
| 30% | 192 (1.39) | 11538 (83.67) | 303 (2.20) | 1757 (12.74) | 14.37 | 97.06 | 55.72 | 32.82 | 91.89 | 4.89 | 0.88 | 19.99 | 16.81 |

Abbreviations: Thr%=developed model’s prediction threshold, TP=true positive, TN=true negative, FP=false positive, FN=false negative, Sens%=sensitivity%, Spec%=specificity%, BAC%=balanced accuracy, PPV%=positive predictive value%, NPV%=negative predictive value%, LR+=positive likelihood ratio, LR-=negative likelihood ratio, F1%=F1 statistic, MCC%=Matthew’s correlation coefficient.

**Supplementary Table 4. Classification Performance of Machine Learning Model Predictions for Two-Year Relapse in Bipolar Disorder Across Varying Threshold Levels in the Finnish External Validation Cohort (N=13,790).**

| **Thr%** | **TP (%)** | **TN (%)** | **FP (%)** | **FN (%)** | **Sens%** | **Spec%** | **BAC%** | **PPV%** | **NPV%** | **LR+** | **LR-** | **F1%** | **MCC%** |
| --- | --- | --- | --- | --- | --- | --- | --- | --- | --- | --- | --- | --- | --- |
| 3% | 1948 (14.13) | 18 (0.13) | 11823 (85.74) | 1 (0.01) | 99.95 | 0.15 | 50.05 | 14.15 | 94.74 | 1.00 | 0.34 | 24.78 | 0.95 |
| 4% | 1624 (11.78) | 3737 (27.10) | 8104 (58.77) | 325 (2.36) | 83.32 | 31.56 | 57.44 | 16.69 | 92.00 | 1.22 | 0.53 | 27.82 | 11.38 |
| 5% | 1521 (11.03) | 4743 (34.39) | 7098 (51.47) | 428 (3.10) | 78.04 | 40.06 | 59.05 | 17.65 | 91.72 | 1.30 | 0.55 | 28.79 | 13.02 |
| 6% | 1364 (9.89) | 6580 (47.72) | 5261 (38.15) | 585 (4.24) | 69.98 | 55.57 | 62.78 | 20.59 | 91.84 | 1.58 | 0.54 | 31.82 | 17.82 |
| 7% | 1283 (9.30) | 7301 (52.94) | 4540 (32.92) | 666 (4.83) | 65.83 | 61.66 | 63.74 | 22.03 | 91.64 | 1.72 | 0.55 | 33.02 | 19.39 |
| 8% | 1171 (8.49) | 8174 (59.27) | 3667 (26.59) | 778 (5.64) | 60.08 | 69.03 | 64.56 | 24.20 | 91.31 | 1.94 | 0.58 | 34.51 | 21.25 |
| 9% | 1105 (8.01) | 8559 (62.07) | 3282 (23.80) | 844 (6.12) | 56.70 | 72.28 | 64.49 | 25.19 | 91.02 | 2.05 | 0.60 | 34.88 | 21.67 |
| 10% | 1068 (7.74) | 8762 (63.54) | 3079 (22.33) | 881 (6.39) | 54.80 | 74.00 | 64.40 | 25.75 | 90.86 | 2.11 | 0.61 | 35.04 | 21.87 |
| 15% | 807 (5.85) | 9950 (72.15) | 1891 (13.71) | 1142 (8.28) | 41.41 | 84.03 | 62.72 | 29.91 | 89.70 | 2.59 | 0.70 | 34.73 | 22.34 |
| 20% | 616 (4.47) | 10657 (77.28) | 1184 (8.59) | 1333 (9.67) | 31.61 | 90.00 | 60.80 | 34.22 | 88.88 | 3.16 | 0.76 | 32.86 | 22.34 |
| 30% | 192 (1.39) | 11538 (83.67) | 303 (2.20) | 1757 (12.74) | 9.85 | 97.44 | 53.65 | 38.79 | 86.78 | 3.85 | 2.10 | 15.71 | 13.66 |

Abbreviations: Thr%=developed model’s prediction threshold, TP=true positive, TN=true negative, FP=false positive, FN=false negative, Sens%=sensitivity%, Spec%=specificity%, BAC%=balanced accuracy, PPV%=positive predictive value%, NPV%=negative predictive value%, LR+=positive likelihood ratio, LR-=negative likelihood ratio, F1%=F1 statistic, MCC%=Matthew’s correlation coefficient.

**Supplementary Table 5. Fairness Analysis of Machine Learning Model Predictions for Relapse in the Swedish Internal Validation Sample**

| **Variable** | **AUROC (95%CI)** | **Statistical Testing (P-value)** | **Brier Score (95%CI)** | **Statistical Testing (P-value)** | **Slope (95%CI)** | **Statistical Testing (P-value)** | **Intercept (95%CI)** | **Statistical Testing (P-value)** |
| --- | --- | --- | --- | --- | --- | --- | --- | --- |
| Education |  |  |  |  |  |  |  |  |
| Elementary Education (N=4163) | 0.72 (0.69-0.73) | 0.783 | 0.079 (0.072-0.085) | 0.994 | 0.961 (0.838-1.083) | 0.742 | -0.104 (-0.382-0.165) | 0.881 |
| >9 Years of Education (N=13,026) | 0.71 (0.69-0.74) |  | 0.075 (0.072-0.078) |  | 0.988 (0.915-1.06) |  | -0.134 (-0.285-0.021) |  |
| Immigration |  |  |  |  |  |  |  |  |
| Refugees/Migrants (N=2390) | 0.70, (0.66-0.73) | 0.575 | 0.085 (0.077-0.094) | 0.980 | 0.889 (0.738-1.06) | 0.455 | -0.259 (-0.626-0.091) | 0.624 |
| Born in Sweden (N=15,675) | 0.71 (0.69-0.72) |  | 0.076 (0.073-0.079) |  | 0.964 (0.898-1.033) |  | -0.151 (-0.295--0.011) |  |
| Gender |  |  |  |  |  |  |  |  |
| Females (N=11,984) | 0.71 (0.68-0.73) | 0.884 | 0.077 (0.073-0.08) | 1.000 | 0.973 (0.895-1.051) | 0.519 | -0.093 (-0.266-0.088) | 0.178 |
| Males (N=6081) | 0.71 (0.69-0.72) |  | 0.079 (0.074-0.084) |  | 0.929 (0.833-1.034) |  | -0.276 (-0.492--0.063) |  |

**Supplementary Figure 1.** **Flowchart Illustrating the Formation of the Swedish and Finnish Cohorts.**


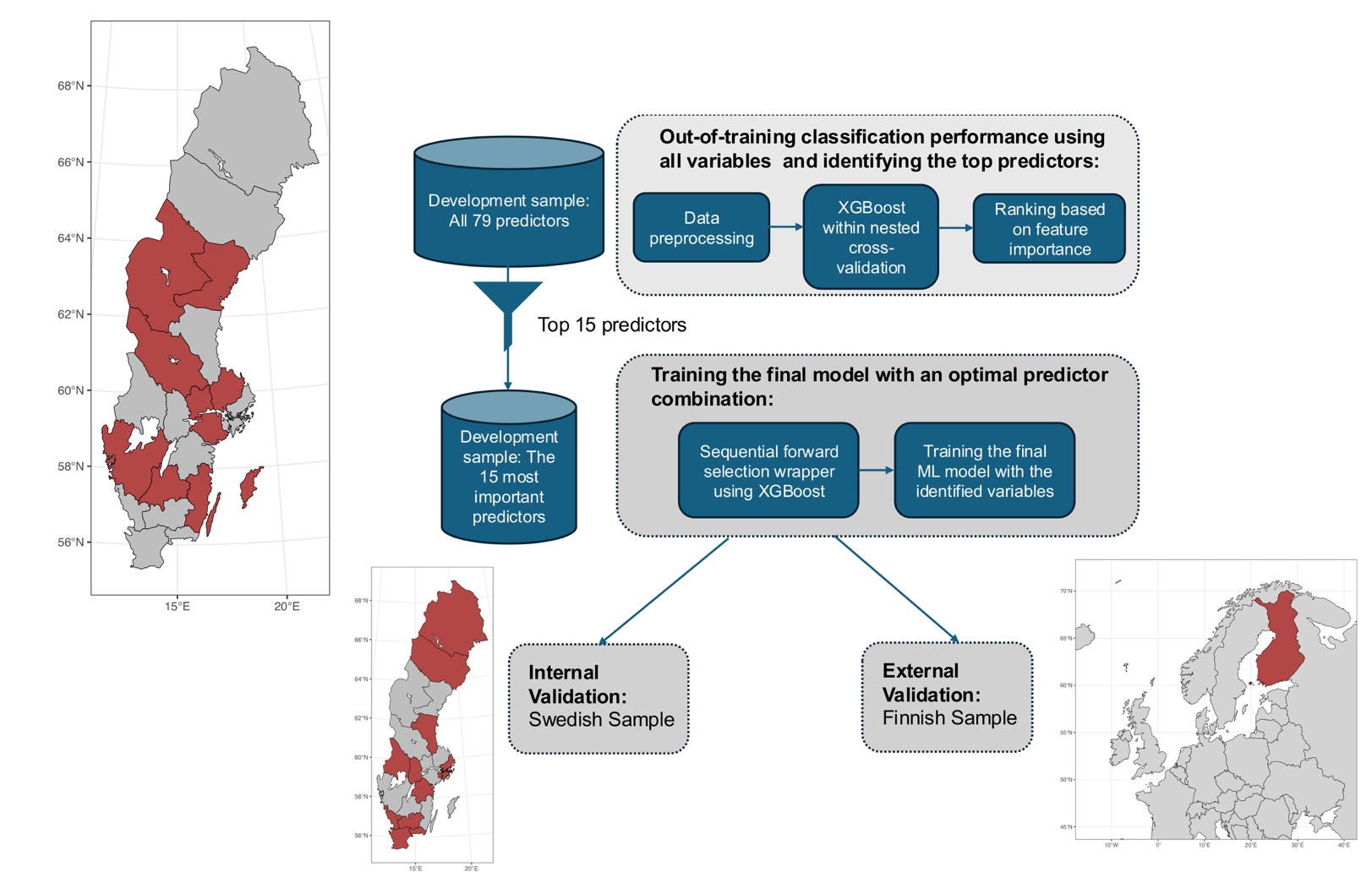


**Supplementary Figure 2.** **Flowchart Illustrating the Development and Validation of the Machine Learning Model in the Swedish and Finnish Cohorts.** Using all 79 candidate predictors in the Swedish development sample (counties in red, top left), the 15 most informative variables were identified and refined via sequential forward search. The final model was trained and internally validated in held-out Swedish counties (in red, bottom left), and externally validated in the Finnish cohort (in red, bottom right).

**
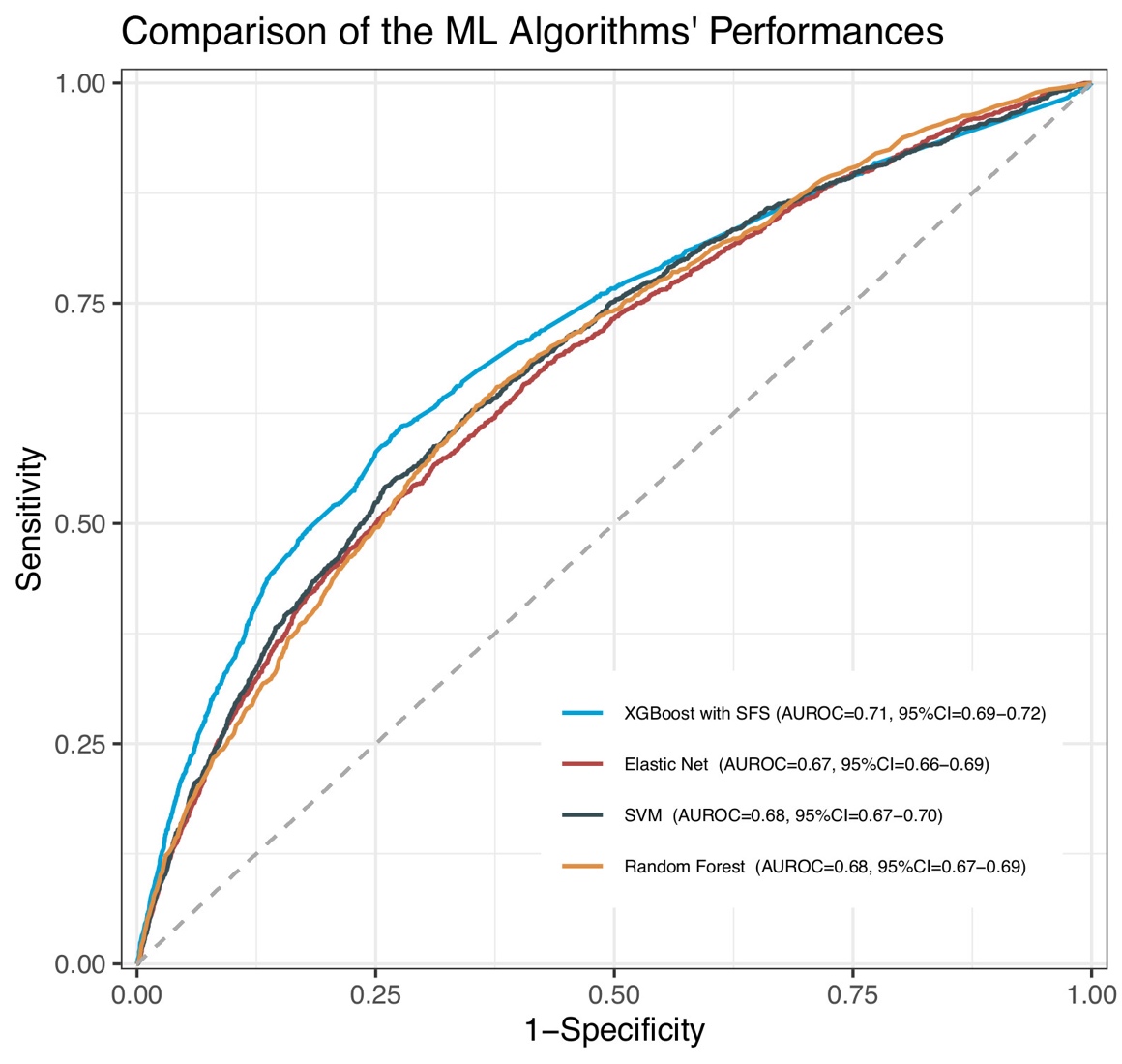
**

**Supplementary Figure 3. Comparison of Discrimination Performance Across Machine Learning Algorithms in the Internal Validation Sample.** The XGBoost model demonstrated superior discrimination performance, as measured by the area under the receiver operating characteristic curve (AUROC), compared with alternative machine learning algorithms (elastic net regression, support vector machine, and random forest): De Long’s tests between other models vs. the XGBoost model: all P-values <0.0001.

**
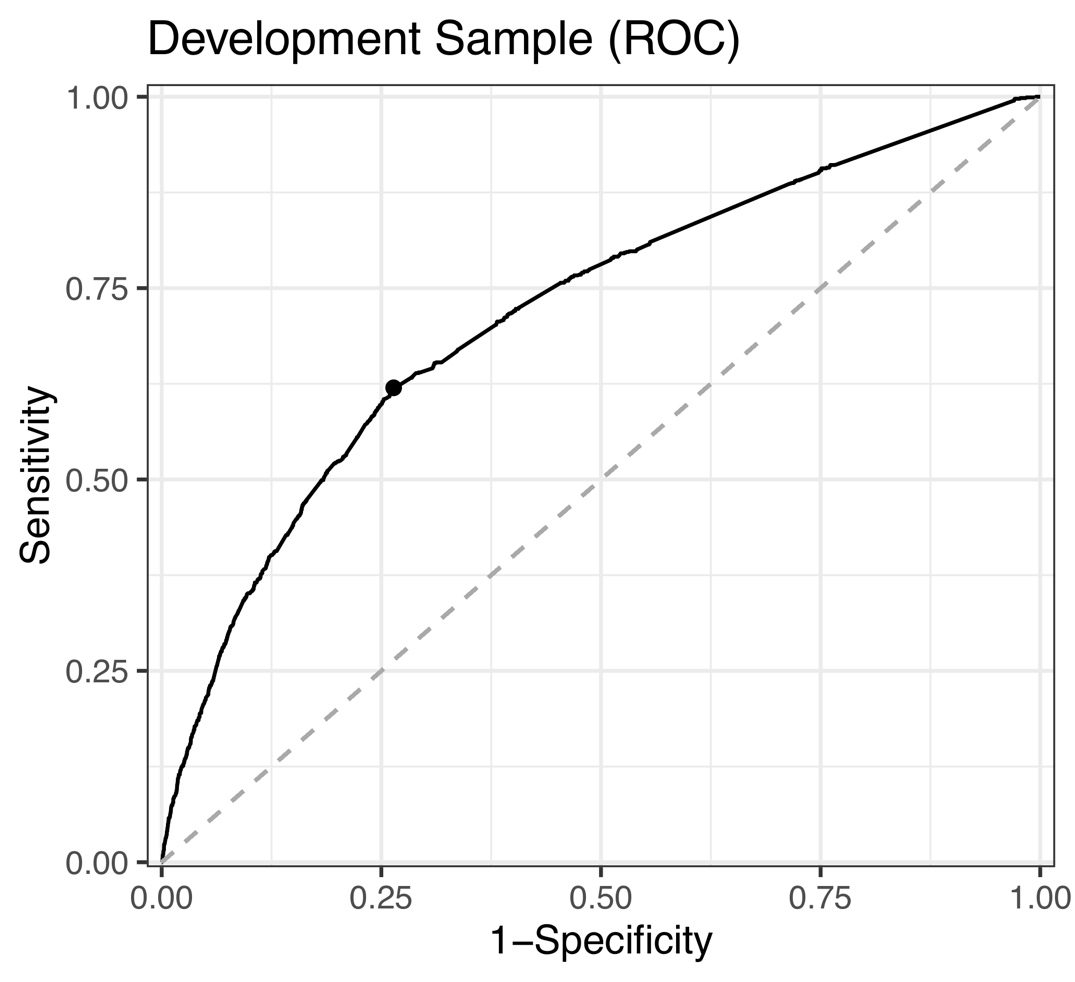
**

**Supplementary Figure 4. Receiver Operating Characteristic (ROC) Curve for the Development Sample.** The ROC curve illustrates model discrimination in the development sample (AUROC 0.71, 95% CI 0.69–0.72). The dot marks the Youden Index, denoting the optimal threshold (predicted risk 9.15%) used to stratify individuals into high- and low-risk groups.

**
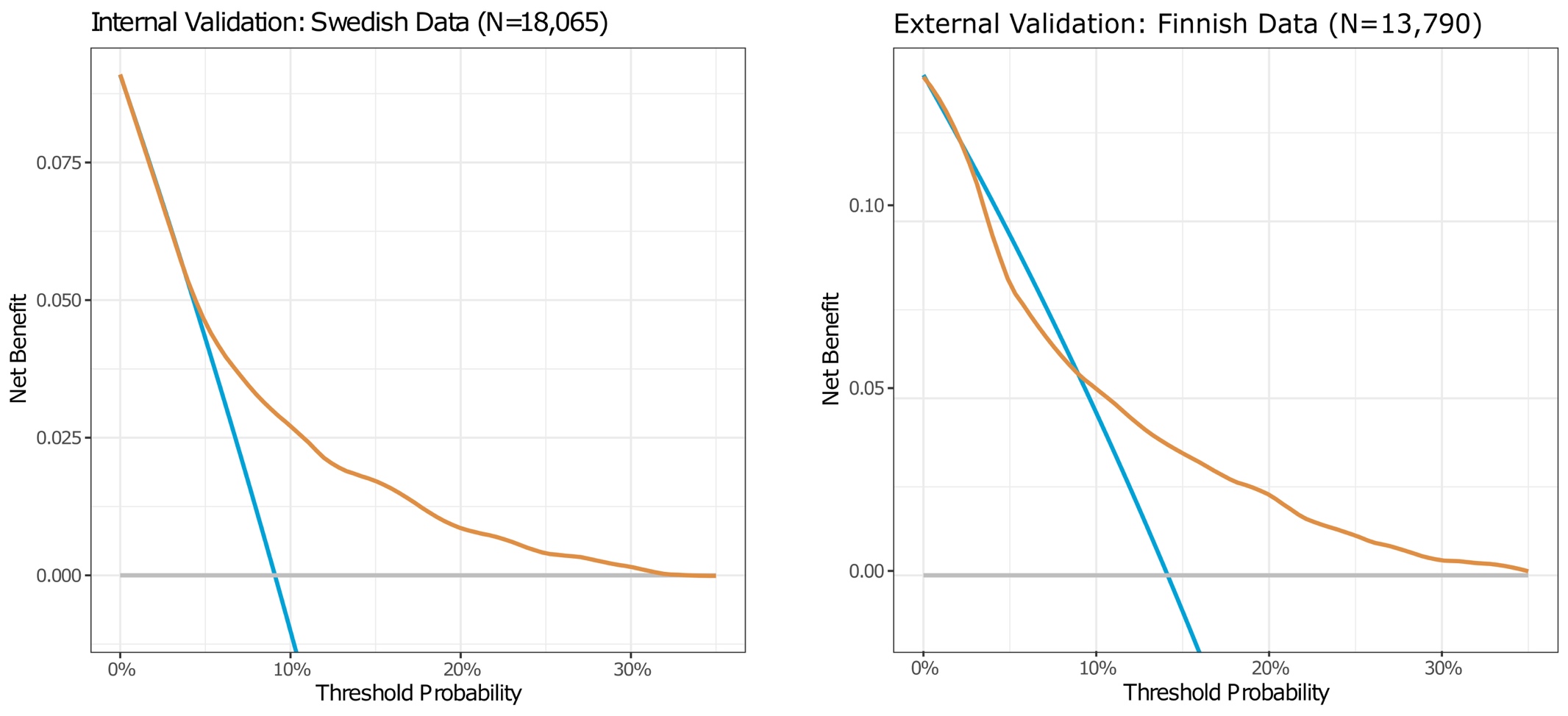
**

**Supplementary Figure 5.** **Decision curve analysis of the developed ML model in predicting two-year bipolar relapse.** The net benefit of the ML model (orange line) is compared with "treat all" (blue line) and "treat none" (gray line) strategies across varying threshold probabilities. (A) Internal validation in the Swedish cohort (N=18,065) shows clinical utility between 4%–34% thresholds. (B) External validation in the Finnish cohort (N=13,790), with net benefit observed between 9%–40% thresholds.


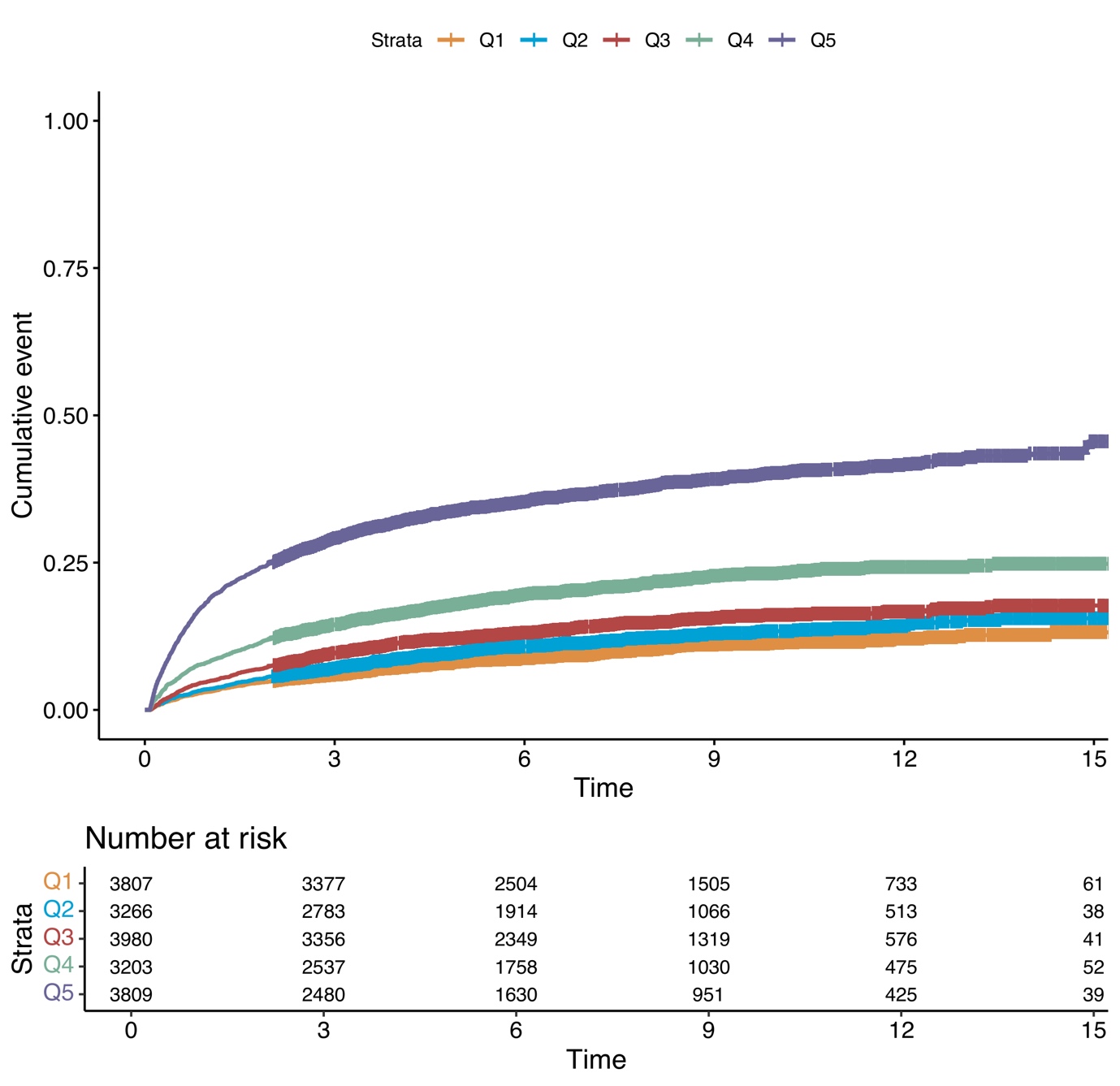


**Supplementary Figure 6.** **Cumulative incidence of relapse by predicted-risk quintile in the Swedish internal validation sample over the 15-year follow-up.** Quintiles were derived from the development sample by using calibrated model-derived probabilities (Q1=0%-3.75%, Q2=3.75%-4.56%, Q3=4.56%-7.85%, Q4=7.85%-13.32%, Q5>13.32%).


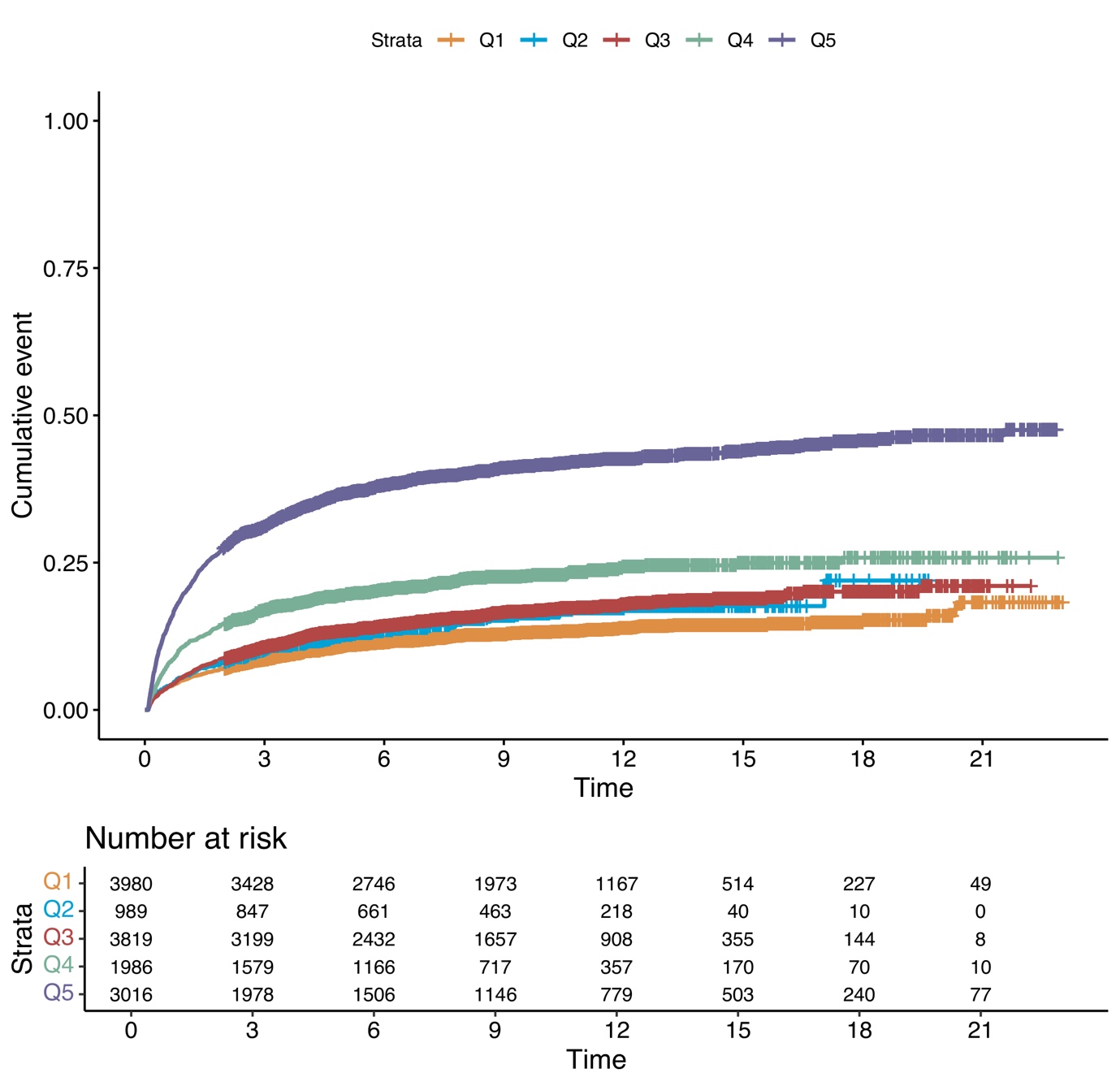


**Supplementary Figure 7.** **Cumulative incidence of relapse by predicted-risk quintile in the Finnish external validation sample over the 23-year follow-up.** Quintiles were derived from the development sample by using calibrated model-derived probabilities (Q1=0%-3.75%, Q2=3.75%-4.56%, Q3=4.56%-7.85%, Q4=7.85%-13.32%, Q5>13.32%).

**
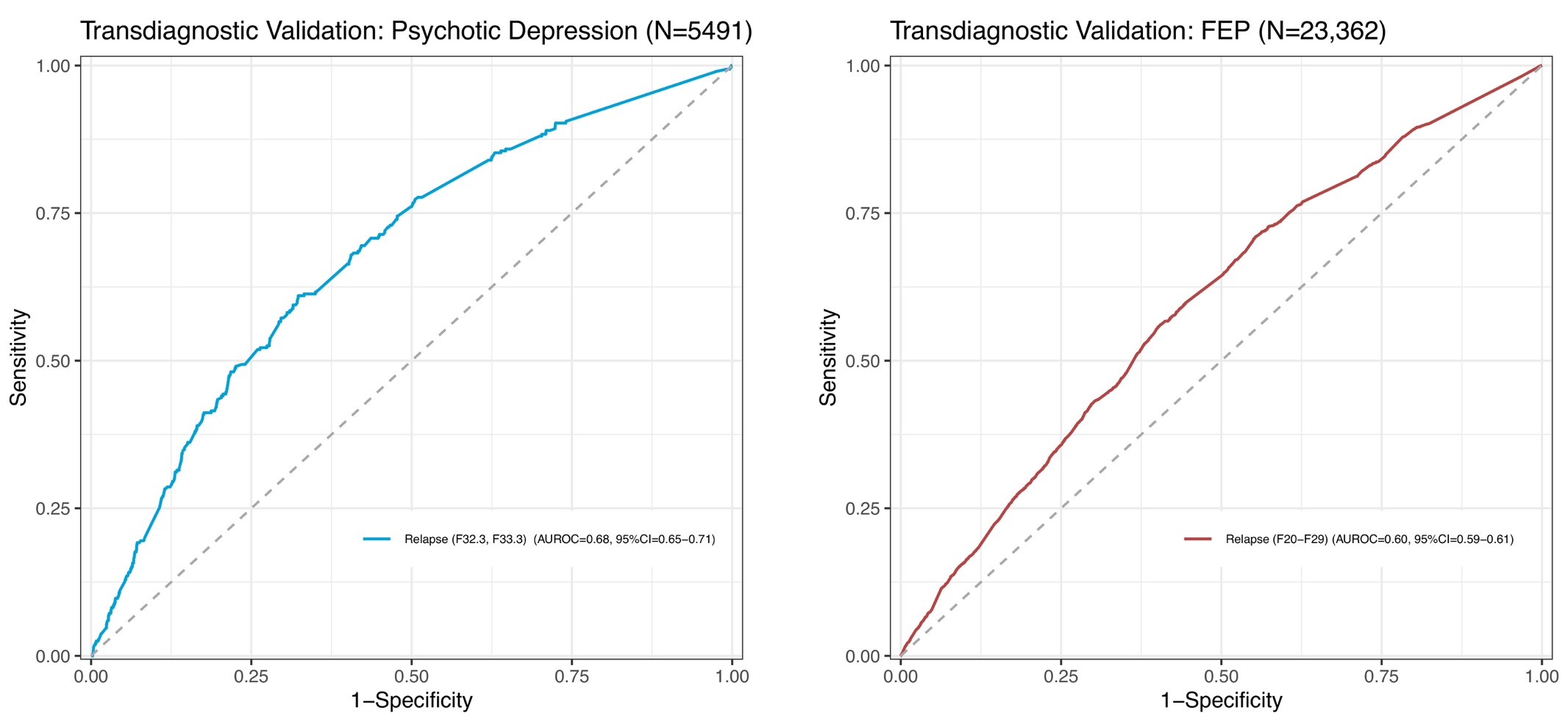
Supplementary Figure 8.** **Discrimination of the Developed Machine Learning Model in First-Episode Psychotic Depression (PD) and First-Episode Non-Affective Psychosis (FEP).**

**
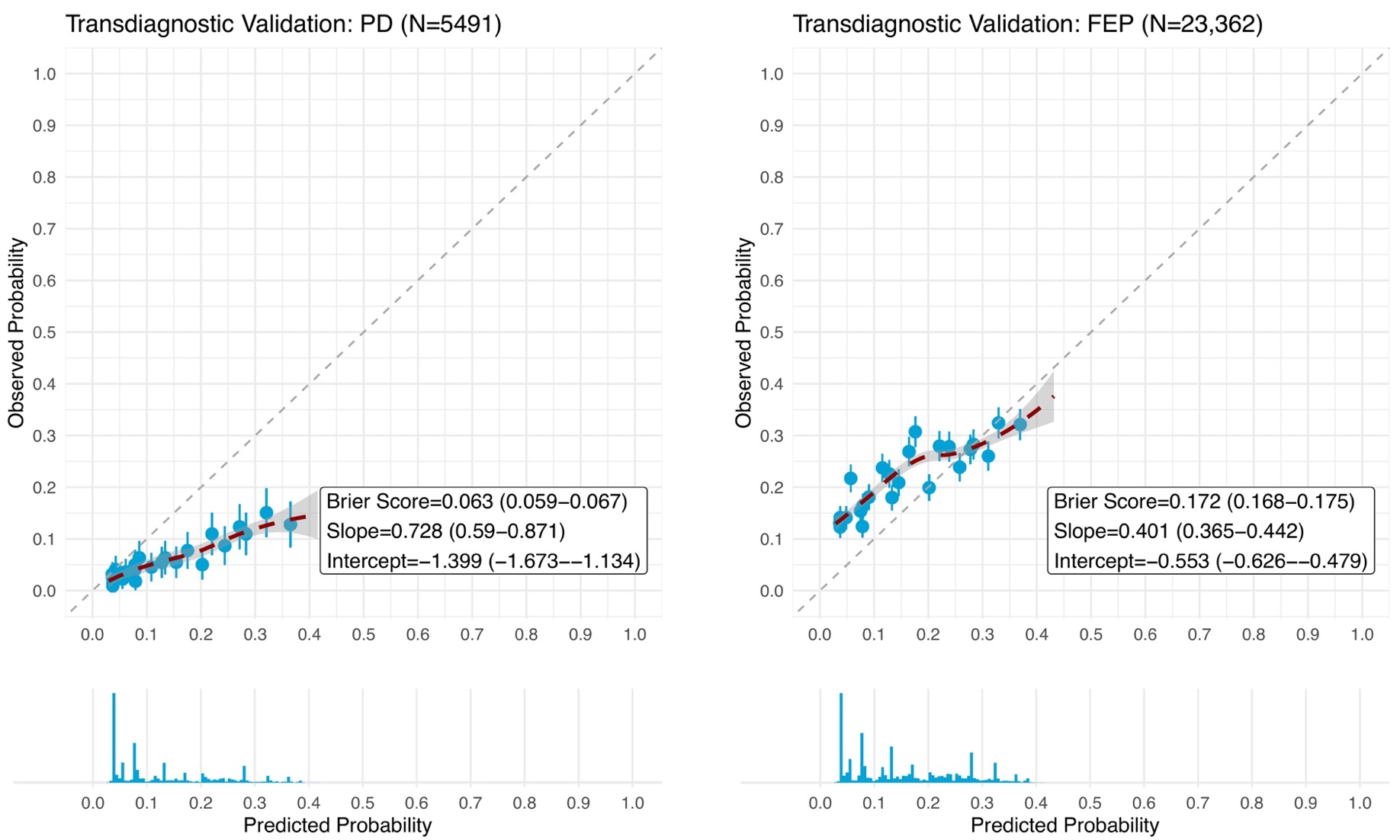
**

**Supplementary Figure 9.** **Calibration Plots of the Developed Machine Learning Model in First-Episode Psychotic Depression (PD) and First-Episode Non-Affective Psychosis (FEP).** Red dashed lines represent smoothed nonlinear curves generated using a loess smoother with 95%CIs (shaded grey). Histograms display the frequency of the model’s probability predictions.

**Supplementary References**

1. Poranen J, Koistinaho A, Tanskanen A, Tiihonen J, Taipale H, Lähteenvuo M. Twenty-year medication use trends in first-episode bipolar disorder. *Acta Psychiatr Scand*. 2022;146(6):583-593. doi:10.1111/acps.13504

2. Koistinaho A, Poranen J, Tanskanen A, Tiihonen J, Taipale H, Lähteenvuo M. Real-world use of pharmacological treatments for incident bipolar disorder: A Finnish nationwide cohort study. *J Affect Disord*. 2023;340:237-244. doi:10.1016/j.jad.2023.08.015

3. Kuhn M. Building Predictive Models in R Using the caret Package. *J Stat Soft*. 2008;28(5):1-26. doi:10.18637/jss.v028.i05

4. Bischl B, Lang M, Kotthoff L, et al. mlr: Machine Learning in R. *The Journal of Machine Learning Research*. 2016;17(1):5938-5942.

5. Chen T, Guestrin C. Xgboost: A scalable tree boosting system. In: ; 2016:785-794.

6. Vickers AJ, van Calster B, Steyerberg EW. A simple, step-by-step guide to interpreting decision curve analysis. *Diagnostic and Prognostic Research*. 2019;3(1):18. doi:10.1186/s41512-019-0064-7
